# Predicting hospital admission at emergency department triage using machine learning

**DOI:** 10.1371/journal.pone.0201016

**Published:** 2018-07-20

**Authors:** Woo Suk Hong, Adrian Daniel Haimovich, R. Andrew Taylor

**Affiliations:** 1 Yale School of Medicine, New Haven, Connecticut, United States of America; 2 Department of Emergency Medicine, Yale School of Medicine, New Haven, Connecticut, United States of America; University of North Texas, UNITED STATES

## Abstract

**Objective:**

To predict hospital admission at the time of ED triage using patient history in addition to information collected at triage.

**Methods:**

This retrospective study included all adult ED visits between March 2014 and July 2017 from one academic and two community emergency rooms that resulted in either admission or discharge. A total of 972 variables were extracted per patient visit. Samples were randomly partitioned into training (80%), validation (10%), and test (10%) sets. We trained a series of nine binary classifiers using logistic regression (LR), gradient boosting (XGBoost), and deep neural networks (DNN) on three dataset types: one using only triage information, one using only patient history, and one using the full set of variables. Next, we tested the potential benefit of additional training samples by training models on increasing fractions of our data. Lastly, variables of importance were identified using information gain as a metric to create a low-dimensional model.

**Results:**

A total of 560,486 patient visits were included in the study, with an overall admission risk of 29.7%. Models trained on triage information yielded a test AUC of 0.87 for LR (95% CI 0.86–0.87), 0.87 for XGBoost (95% CI 0.87–0.88) and 0.87 for DNN (95% CI 0.87–0.88). Models trained on patient history yielded an AUC of 0.86 for LR (95% CI 0.86–0.87), 0.87 for XGBoost (95% CI 0.87–0.87) and 0.87 for DNN (95% CI 0.87–0.88). Models trained on the full set of variables yielded an AUC of 0.91 for LR (95% CI 0.91–0.91), 0.92 for XGBoost (95% CI 0.92–0.93) and 0.92 for DNN (95% CI 0.92–0.92). All algorithms reached maximum performance at 50% of the training set or less. A low-dimensional XGBoost model built on ESI level, outpatient medication counts, demographics, and hospital usage statistics yielded an AUC of 0.91 (95% CI 0.91–0.91).

**Conclusion:**

Machine learning can robustly predict hospital admission using triage information and patient history. The addition of historical information improves predictive performance significantly compared to using triage information alone, highlighting the need to incorporate these variables into prediction models.

## Introduction

While most emergency department (ED) visits end in discharge, EDs represent the largest source of hospital admissions [1]. Upon arrival to the ED, patients are first sorted by acuity in order to prioritize individuals requiring urgent medical intervention. This sorting process, called "triage", is typically performed by a member of the nursing staff based on the patient's demographics, chief complaint, and vital signs. Subsequently, the patient is seen by a medical provider who creates the initial care plan and ultimately recommends a disposition, which this study limits to hospital admission or discharge.

Prediction models in medicine seek to improve patient care and increase logistical efficiency [2,3]. For example, prediction models for sepsis or acute coronary syndrome are designed to alert providers of potentially life-threatening conditions, while models for hospital utilization or patient-flow enable resource optimization on a systems level [4–8]. Early identification of ED patients who are likely to require admission may enable better optimization of hospital resources through improved understanding of ED patient mixtures [9]. It is increasingly understood that ED crowding is correlated with poorer patient outcomes [10]. Notification of administrators and inpatient teams regarding potential admissions may help alleviate this problem [11]. From the perspective of patient care in the ED setting, a patient's likelihood of admission may serve as a proxy for acuity, which is used in a number of downstream decisions such as bed placement and the need for emergency intervention [12–14].

Numerous prior studies have sought to predict hospital admission at the time of ED triage. Most models only include information collected at triage such as demographics, vital signs, chief complaint, nursing notes, and early diagnostics [11,14–19], while some models include additional features such as hospital usage statistics and past medical history [9,12,20,21]. A few models built on triage information have been formalized into clinical decision rules such as the Sydney Triage to Admission Risk Tool and the Glasgow Admission Prediction Score [22–25]. Notably, a progressive modeling approach that uses information available at later time-points, such as lab tests ordered, medications given, and diagnoses entered by the ED provider during the patient’s current visit, has been able to achieve high predictive power and indicates the utility of these features [20,21]. We hypothesized that extracting such features from a patient’s previous ED visits would lead to a robust model for predicting admission at the time of triage. Prior models that incorporate past medical history utilize simplified chronic disease categories such as heart disease or diabetes [9,12] while leaving out rich historical information accessible from the electronic health record (EHR) such as outpatient medications and historical labs and vitals, all of which are routinely reviewed by providers when evaluating a patient. As a recent work showed that using all elements of the electronic health record can robustly predict in-patient outcomes [26], a prediction model for admission built on comprehensive elements of patient history may improve on prior models.

Furthermore, many prior studies have been limited by technical factors, where continuous variables are often reduced to categorical variables through binning or to binary variables encoding presence or missing-ness of data due to the challenges of imputation [9,15,16,19–21]. Logistic regression and Naive Bayes are commonly used [9,11,16,18–22], with few studies using more complex algorithms like random-forests, artificial neural networks, and support vector machines [12,15,17]. While gradient boosting and deep neural networks have been shown to be powerful tools for predictive modeling, neither has been applied to the task of predicting admission at ED triage to date.

Expanding on prior work [9,12,20], we build a series of binary classifiers on 560,486 patient visits, with 972 variables extracted per visit from the EHR, including previous healthcare usage statistics, past medical history, historical labs and vitals, prior imaging counts, and outpatient medications, as well as fine demographic details such as insurance and employment status. We use gradient boosting and deep neural networks, two of the best performing algorithms in classification tasks, to model the nonlinear relationships among these variables. Moreover, we test whether we have achieved maximum performance for our feature set by measuring performance across models trained on increasing fractions of our data. Lastly, we identify variables of importance using information gain as our metric and present a low-dimensional model amenable to implementation as clinical decision support.

## Materials and methods

### Study setting

Retrospective data was obtained from three EDs covering the period of March 2013 to July 2017 to ensure a 1-year of historical timeframe from the study start period of March 2014. The represented EDs include a level I trauma center with an annual census of approximately 85,000 patients, a community hospital-based department with an annual census of approximately 75,000 patients, and a suburban, free-standing department with an annual census of approximately 30,000 patients. All three EDs are part of a single hospital system utilizing the Epic EHR (Verona, WI) and the Emergency Severity Index (ESI) for triage. The study included all visits for adult patients with a clear, recorded disposition of either admission or discharge. Individuals with any other disposition, such as transfer, AMA, and eloped, were excluded. This study was approved, and the informed consent process waived, by the Yale Human Investigation Committee (IRB 2000021295).

### Data collection and processing

For each patient visit, we collected a total of 972 variables, divided into major categories shown in Table 1. The full list of variables is provided in S1 Table. All data elements were obtained from the enterprise data warehouse, using SQL queries to extract relevant raw-data in comma-separated value format. All subsequent processing was done in R. The link to a repository containing the de-identified dataset and R scripts are available in S1 Text. Below, we summarize the processing steps for each category.

**Table 1:** Variables included in models.

Category	Number of Variables	Only Triage	Only History	Full
Response variable (Disposition)	1	X	X	X
Demographics	9	X	X	X
Triage evaluation	13	X		X
Chief complaint	200	X		X
Hospital usage statistic	4		X	X
Past medical history	281		X	X
Outpatient medications	48		X	X
Historical vitals	28		X	X
Historical labs	379		X	X
Imaging/EKG counts	9		X	X
Total	972	223	759	972

Only Triage—model using only triage information. Only History—model using only patient history. Full—model using the full set of variables. Note that demographic information is included in all three models.

#### Response variable

The primary response variable was the patient's disposition, encoded in a binary variable (1 = admission, 0 = discharge).

#### Demographics

Demographic information, either collected at triage or available from EHR at the time of patient encounter, included age, gender, primary language, ethnicity, employment status, insurance status, marital status, and religion. The primary language variable was recoded into a binary split (e.g., English vs. non-English), while the top twelve levels comprising >95% of all visits were retained for the religion variable and all other levels binned to one 'Other' category. All unique levels were retained for other demographic variables.

#### Triage evaluation

Triage evaluation included variables routinely collected at triage, such as the name of presenting hospital, arrival time (month, day, 4-hr bin), arrival method, triage vital signs, and ESI level assigned by the triage nurse. Triage vital signs included systolic and diastolic blood pressure, pulse, respiratory rate, oxygen saturation, presence of oxygen device, and temperature. Values beyond physiologic limits were replaced with missing values.

#### Chief complaint

Given the high number of unique values (> 1000) for chief complaint, the top 200 most frequent values, which comprised >90% of all visits, were retained as unique categories and all other values binned into 'Other'.

#### Hospital usage statistic

The number of ED visits within one year, the number of admissions within one year, the disposition of the patient's previous ED visit, and the number of procedures and surgeries listed in the patient's record at the time of encounter were taken as metrics for prior hospital usage.

#### Past medical history

ICD-9 codes for past medical history (PMH) were mapped onto 281 clinically meaningful categories using the Agency for Healthcare Research and Quality (AHRQ) Clinical Classification Software (CCS), such that each CCS category became a binary variable with the value 1 if the patient's PMH contained one or more ICD-9 code belonging in that category and 0 otherwise.

#### Outpatient medications

Outpatient medications listed in the EHR as active at the time of patient encounter were binned into 48 therapeutic subgroups (e.g. cardiovascular, analgesics) used internally by the Epic EHR system, with each corresponding variable representing the number of medications in that subgroup.

#### Historical vitals

A time-frame of one year from the date of patient encounter was used to calculate historical information, which included vital signs, labs and imaging previously ordered from any of the three EDs. Historical vital signs were represented by the minimum, maximum, median, and the last recorded value of systolic blood pressure, diastolic blood pressure, pulse, respiratory rate, oxygen saturation, presence of oxygen device, and temperature. Values beyond physiologic limits were replaced with missing values.

#### Historical labs

Given the diversity of labs ordered within the ED, the 150 most frequent labs comprising 94% of all orders were extracted then divided into labs with numeric values and those with categorical values. The cutoff of 150 was chosen to include labs ordered commonly enough to be significant in the management of most patients (e.g., Troponin T, BNP, CK, D-Dimer), even if they were not as frequent as routine labs like CBC, BMP, and urinalysis. The minimum, maximum, median, and the last recorded value of each numeric lab were included as features. Categorical labs, which included urinalysis and culture results, were recoded into binary variables with 1 for any positive value (e.g. positive, trace, +, large) and 0 otherwise. Any growth in blood culture was labeled positive as were urine cultures with > 49,000 colonies/mL. The number of tests, the number of positives, and the last recorded value of each categorical lab were included as features.

#### Imaging and EKG counts

The number of orders were counted for each of the following categories: electrocardiogram (EKG), chest x-ray, other x-ray, echocardiogram, other ultrasound, head CT, other CT, MRI, and all other imaging.

### Model fitting and evaluation

A series of nine binary classifiers were trained using logistic regression (LR), gradient boosting (XGBoost), and deep neural networks (DNN) on three dataset types: one using only triage information, one using only patient history, and one using the full set of variables (Table 1). All analyses were done in R using the caret, xgboost, and keras packages [27–29].

A randomly chosen test set of 56,000 (10%) samples was held out, then the remaining 504,486 (90%) samples split randomly five times to create five independent validation sets of 56,000 (10%) and training sets of 448,486 (80%). Hyperparameters for each model were optimized by maximizing the average validation AUC across the five validation sets. The optimized set of hyperparameters was then used to train the model on all 504,486 (90%) samples excluding the test set. Finally, the test AUC was calculated on the held-out test set, with 95% confidence intervals constructed using the DeLong method implemented in the pROC package [30,31]. Youden's index was used to find the optimal cutoff point on the ROC curve to calculate the sensitivity, specificity, positive predictive value, and negative predictive value for each model [32,33]. Details of the tuning process are provided in S1 Text.

Categorical variables were converted into binary variables prior to training using one-hot encoding [34]. The median for each variable post normalization was used to impute the input matrix for LR and DNN. The sparsity of our dataset prevented taking a more sophisticated imputation approach such as k-nearest neighbors or random forests [35,36]. An alternative to imputation is to transform all continuous variables into categorical variables, binning NA into a separate category, then performing one-hot encoding [37]. However, this approach loses all ordinal information and thus was not taken. Imputation was not performed for XGBoost, since the algorithm learns a default direction for each split in the case that the variable needed for the split is missing [28].

### Testing the benefit of additional training samples

One key question in predictive modeling is whether additional training samples will improve performance or whether a model has reached its maximum performance given the inherent noise in its features [38]. To test the potential benefit of additional training samples, we trained full-variable models using each of the three algorithms on randomly selected fractions of the training set (1%, 10%, 30%, 50%, 80%, 100%), then calculated their AUCs on the held-out test set in order to quantify the incremental gain in performance.

### Variables of importance

Information gain is a metric that quantifies the improvement in accuracy of a tree-based algorithm from a split based on a given variable [39]. We calculated the mean information gain for each variable based on 100 training iterations of the full XGBoost model. We then trained a low-dimensional XGBoost model using a subset of variables with high information gain to test whether such a model could predict hospital admission as robustly as the full model.

## Results

### Characteristics of study samples

A total of 560,486 ED visits were available for analysis after filtering for exclusion criteria, with 13% of the samples excluded due to disposition other than admission or discharge. The visits represented 202,953 unique patients, with a median visit count of 1 and a mean visit count of 2.76 per patient during the study duration. The overall hospital admission risk was 29.7% and decreased by triage level: ESI-1 85.6%, ESI-2 55.0%, ESI-3 29.1%, ESI-4 2.2%, and ESI-5 0.4% (S1 Fig). Characteristics of the study samples are presented in Table 2.

**Table 2:** Characteristics of study samples.

	Admitted (n = 166,638)	Discharged (n = 393,848)
Age in mean years (95% CI)	61.6 (61.5–61.7)	44.9 (44.9–45.0)
Gender—Male (%)	77,093 (46.3%)	173,740 (44.1%)
Language—English (%)	154,831 (92.9%)	359,985 (91.4%)
Arrival mode—Ambulance (%)	89,955 (54.0%)	100,415 (25.5%)
Mean triage heart rate (95% CI)	88.9 (88.7–89.0)	84.6 (84.5–84.6)
Mean triage systolic blood pressure (95% CI)	134.7 (134.6–134.9)	132.9 (132.9–133.0)
Mean triage diastolic blood pressure (95% CI)	79.4 (79.3–79.5)	80.8 (80.8–80.9)
Mean triage respiratory rate (95% CI)	18.0 (18.0–18.0)	17.5 (17.5–17.5)
Mean triage oxygen saturation (95% CI)	96.6 (96.6–96.7)	97.5 (97.5–97.5)
Mean triage temperature (95% CI)	98.2 (98.2–98.2)	98.1 (98.1–98.1)
Median ESI Level	2	3

All comparisons were significant with p < 2.2e-16

### Model performance

**Fig 1:**
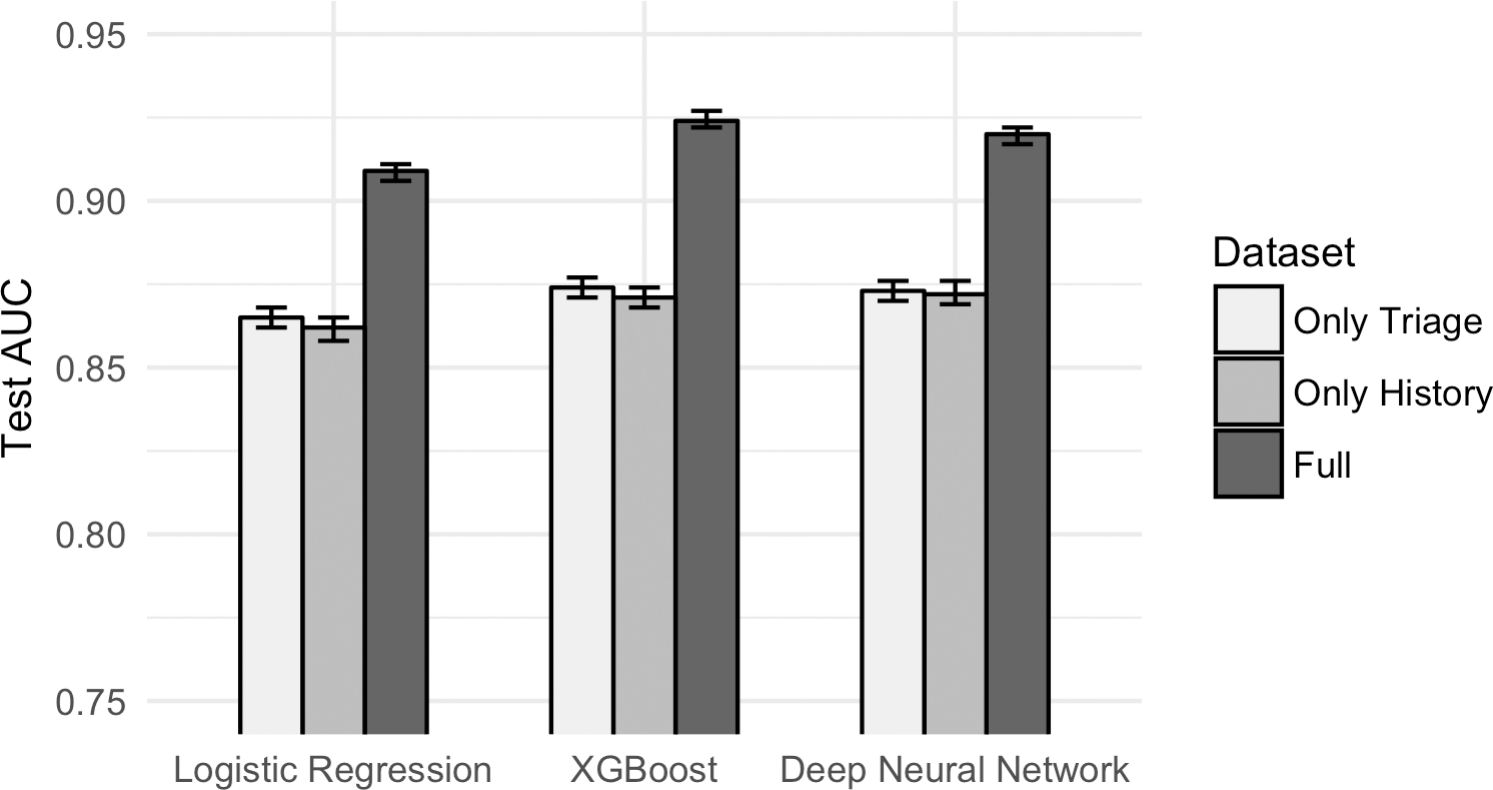
Test AUC by dataset type by algorithm. Addition of historical information improves predictive performance significantly compared to using triage information alone. Patient history alone can predict admission to a reasonable degree.

Models trained on triage information yielded a test AUC of 0.87 for LR (95% CI 0.86–0.87), 0.87 for XGBoost (95% CI 0.87–0.88) and 0.87 for DNN (95% CI 0.87–0.88). Models trained on patient history yielded an AUC of 0.86 for LR (95% CI 0.86–0.87), 0.87 for XGBoost (95% CI 0.87–0.87) and 0.87 for DNN (95% CI 0.87–0.88). Models trained on the full set of variables yielded an AUC of 0.91 for LR (95% CI 0.91–0.91), 0.92 for XGBoost (95% CI 0.92–0.93) and 0.92 for DNN (95% CI 0.92–0.92). The addition of historical information improved predictive performance significantly compared to using triage information alone (Fig 1). Notably, we were able to achieve an AUC of over 0.86 by using patient history alone, which excludes triage level. XGBoost and DNN outperformed LR on the full dataset, while there was no significant difference in performance between XGBoost and DNN across all three dataset types. The sensitivity, specificity, positive-predictive-value (PPV), and negative-predictive-value (NPV) of each model are shown in Table 3. The optimized hyperparameters for each model, as well as its the training and validation AUCs, are provided in S1 Text.

**Table 3:** Summary of statistical measures for each model.

Algorithm	Dataset	Test AUC (95% CI)	Sensitivity	Specificity	PPV	NPV
LR	Only Triage	0.865 (0.862–0.868)	0.68	0.85	0.65	0.87
LR	Only History	0.862 (0.858–0.865)	0.72	0.85	0.67	0.88
LR	Full	0.909 (0.906–0.911)	0.80	0.85	0.69	0.91
XGBoost	Only Triage	0.874 (0.871–0.877)	0.69	0.85	0.66	0.87
XGBoost	Only History	0.871 (0.868–0.874)	0.73	0.85	0.67	0.88
XGBoost	Full	0.924 (0.922–0.927)	0.83	0.85	0.70	0.92
DNN	Only Triage	0.873 (0.870–0.876)	0.70	0.85	0.66	0.87
DNN	Only History	0.872 (0.869–0.876)	0.74	0.85	0.67	0.89
DNN	Full	0.920 (0.917–0.922)	0.82	0.85	0.70	0.92
XGBoost	Top Variables	0.910 (0.908–0.913)	0.79	0.85	0.69	0.91

95% CI for all measures < ± 0.01. The cutoff threshold for each model was set to match a fixed specificity of 0.85 to facilitate comparison. The value of 0.85 was chosen by using Youden’s Index on the full XGBoost model. Models achieving a test AUC greater than 0.9 are shaded in gray.

### Testing the benefit of additional training samples

**Fig 2:**
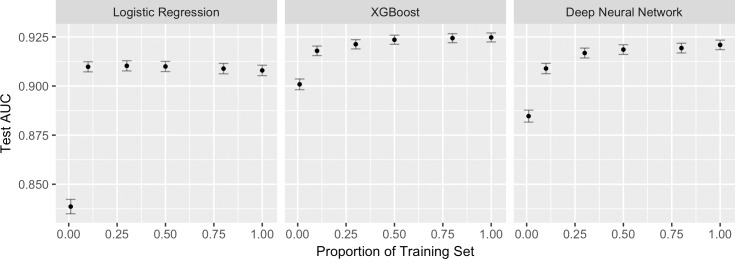
Model performance on increasing fractions of the training set. 95% CIs are shown in gray bars. All three algorithms reach maximum performance at 50% of the training set or less. LR reaches maximum performance earlier than XGBoost or DNN.

For LR, the 95% CI of the AUC of the model trained on 10% of the training set contained the AUC of the model trained on the entire training set. For XGBoost and DNN, the point at which this occurred was at 50% of the training set (Fig 2). All AUC values are provided in S1 Text.

### Variables of importance

**Fig 3:**
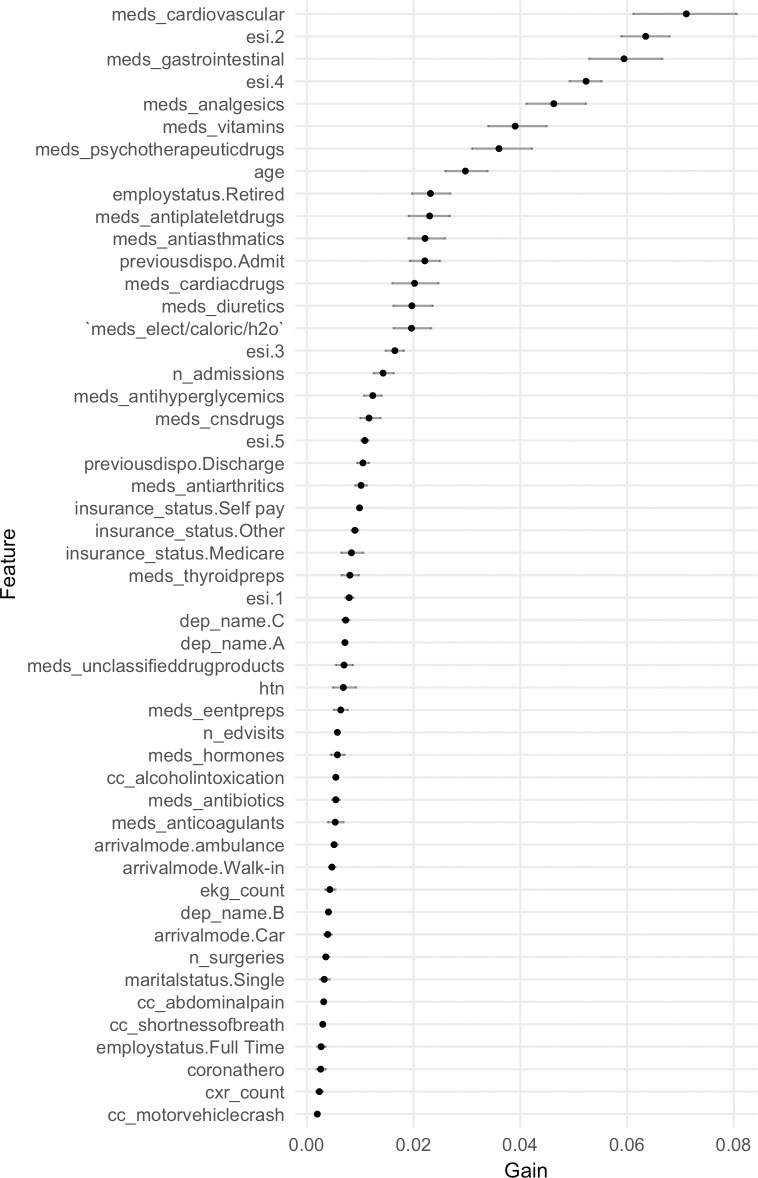
Variables from the full XGBoost model ordered by information gain. Row names represent the variables in the design matrix post one-hot encoding (see S1 Table for name descriptions). Points represent the mean information gain from a hundred runs of XGBoost. Horizontal lines show bootstrapped 95% confidence intervals.

Variables of importance extracted from a hundred iterations of the full XGBoost model are shown in Fig 3 (numeric values provided in S2 Table). Variables representing ESI level, outpatient medication counts, demographics, and hospital usage statistics showed high information gain. A low-dimensional XGBoost model built on variables from these four categories, which include ESI level, age, gender, marital status, employment status, insurance status, race, ethnicity, primary language, religion, number of ED visits within 1 year, number of admissions within 1 year, disposition of the previous ED visit, total number of prior surgeries or procedures, and outpatient medication counts by therapeutic category, yielded a test AUC of 0.91 (95% CI 0.91–0.91).

## Discussion

We describe a series of prediction models for hospital admission that leverage gradient boosting and deep neural networks on a dataset of 560,486 patient visits. Our study shows that machine learning can robustly predict hospital admission at emergency department (ED) triage and that the addition of patient history improves predictive performance significantly compared to using triage information alone, highlighting the need to incorporate these variables into predictive models.

In order to test whether additional training samples will improve performance, we train our models on increasing fractions of the training set and show that the AUC plateaus well below the full training set. This result suggests that we have likely maximized the discriminatory capability for our feature set. More studies will be required to develop features that may further improve performance. We expect that many of these features will be derived from free-text data in the electronic health record (EHR). Specifically, natural language processing of medical notes may provide an informative set of features that capture information absent in tabular data. Recent prediction models on outcomes ranging from sepsis to suicide have demonstrated success with these approaches [4,26,40,41].

The ranking of information gain extracted from the gradient boosting (XGBoost) model present a number of notable features. In particular, the importance of medication counts in the model may either reflect a proxy feature for medical complexity or indicate that polypharmacy itself is a risk factor [42,43]. Not surprisingly, the triage level encoded by the Emergency Severity Index (ESI) had high information gain [44,45], as did prior hospital usage statistics such as the number of admissions within the past year and the disposition of the previous ED visit. Variables correlated with age and markers of socioeconomic status such as insurance type were some of the other features identified by our model that have been previously linked to hospital admission [46–48]. We show that these features can be combined to create a low-dimensional model amenable to implementation in EHR systems as clinical decision support.

This study has a number of limitations. We chose to restrict patient history to information gathered from previous ED visits and anticipate that expanding the sources of historical data may improve model performance. Importing historical data from outpatient clinics or inpatient wards may present technical difficulties regarding EHR integration and differing standards of care. Furthermore, the data utilized in this study came from a hospital system that includes multiple emergency departments with a large catchment area. We anticipate difficulties extending this study to datasets from dense urban areas with multiple independent EDs, given that patients may not consistently present to the same hospital system. Ongoing progress with inter-system information sharing presents one path forward, and this study highlights the importance of those efforts [49,50].

Throughout this study, we predict patient disposition by using the ED provider's prior decision as our true label. In doing so, we are unable to address the appropriateness of individual clinical decisions. This and similar studies would benefit from further research into a gold-standard metric for hospital admission. Studies have suggested that such a metric will remain elusive [51,52]. However, we expect that future work will align response variables of interest with patient-oriented outcomes to create a standardized metric for hospital admission. Future studies may try to adjust the response variable for discharged patients who returned to the ED the next day to get admitted and for admitted patients who in retrospect did not require admission.

Lastly, this study does not address the implementation and efficacy barriers present in clinical practice [53]. While we propose a low-dimensional model with the explicit intent of facilitating implementation into an EHR system, there is no uniform method by which clinical decision support tools are implemented. More systems-based research will be required to analyze methods of implementation and its effect on patient outcomes, with the ultimate goal of providing a standardized evaluation metric for prediction models.

## Supporting information

S1 Table Full list of variables. Descriptions of all 972 variables included in the study.
(XLSX)

S2 Table Variables ranked by information gain. Mean information gain for top variables from 100 runs of XGBoost.
(XLSX)

S1 Fig Patient census by ESI level. (TIF)

S1 Text Model fitting protocol. Details on the model fitting process, including the link to the de-identified dataset and R scripts.
(DOCX)
