# Supplementary figures and images for: Predicting hospital admission at emergency department triage using machine learning
Source: PLoS One. 2018 Jul 20;13(7):e0201016. doi: 10.1371/journal.pone.0201016 (PMC6054406; doi:10.1371/journal.pone.0201016)

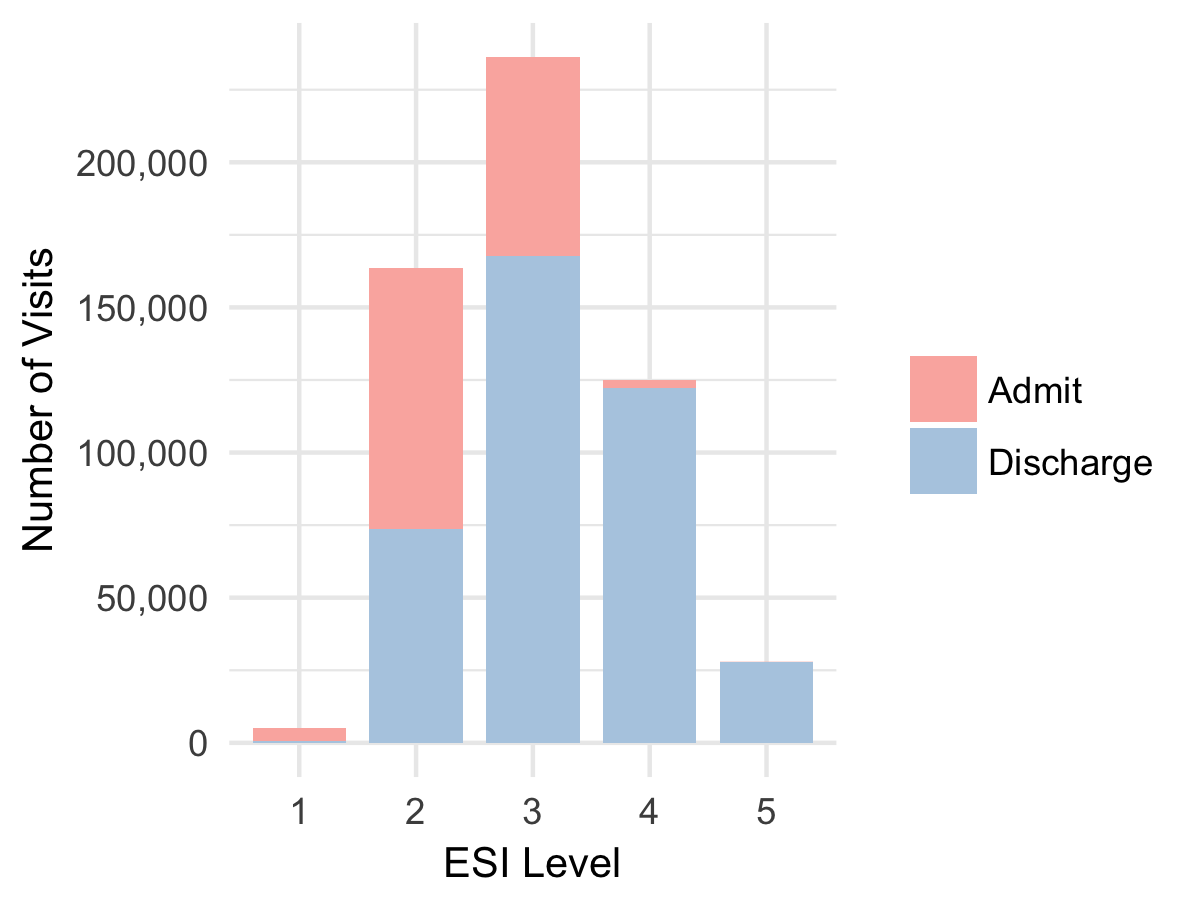

Supplement: S1 Fig — (TIF) [file pone.0201016.s003.tif]
