# Supplementary material for: Predicting hospital admission at emergency department triage using machine learning
Source: PLoS One. 2018 Jul 20;13(7):e0201016. doi: 10.1371/journal.pone.0201016 (PMC6054406; doi:10.1371/journal.pone.0201016)
Supplement: S1 Text — Details on the model fitting process, including the link to the de-identified dataset and R scripts. (DOCX) [file pone.0201016.s004.docx]

**S1 Text: Model fitting protocol**

De-identified dataset, processing and analysis scripts are available in the repository:

<https://github.com/yaleemmlc/admissionprediction>

**Description of algorithms and model tuning process**

**XGBoost** is a scalable implementation of gradient boosting, an algorithm that minimizes loss by combining a series of simple linear or tree-based learners [1,2]. XGBoost has a natural way of handling missing values by learning a default direction in the case that the feature needed for the split is missing, and thus is particularly suited for EHR data. The hyperparameters for the *xgboost* package (version 0.6-4) and their default values are listed in its online documentation (<http://xgboost.readthedocs.io/en/latest/parameter.html>). The following hyperparameters were tuned to maximize the average performance on the validation sets:

- max_depth
- colsample_bylevel
- nrounds

The following hyperparameters were fixed:

- eta = 0.3
- nthread = 5
- eval_metric = 'auc'
- objective = 'binary:logistic'

All other hyperparameters were left to their default values. In case of a tie up to the 3rd decimal place, preference was given to the lower-complexity model. The optimized set of hyperparameters for each XGBoost model is provided in Table A.

**Table A. Hyperparameters for XGBoost models**

| **Algorithm** | **Dataset** | **Hyperparameters** | **Training AUC** | **Val AUC** | **Test AUC** | **95% CI Lower** | **95% CI Upper** |
| --- | --- | --- | --- | --- | --- | --- | --- |
| XGBoost | Only Triage | max_depth = 20, nrounds = 30,  colsample_by_level = 0.1 | 0.901 | 0.874 | 0.874 | 0.871 | 0.877 |
| XGBoost | Only History | max_depth = 20, nrounds = 30,  colsample_by_level = 0.05 | 0.892 | 0.874 | 0.871 | 0.868 | 0.874 |
| XGBoost | Full | max_depth = 20, nrounds = 30,  colsample_by_level = 0.05 | 0.942 | 0.925 | 0.924 | 0.922 | 0.927 |
| XGBoost | Top Variables | max_depth = 10, nrounds = 20,  colsample_by_level = 0.5 | 0.919 | 0.912 | 0.91 | 0.908 | 0.913 |

**Deep neural networks (DNN)**, also known as "deep-learning", are artificial neural networks with several hidden layers where the output of each layer consists of a nonlinear transformation of the weighted sums of the previous layer's outputs [3,4]. Given that EHR data does not have a spatial structure and because our processed inputs included no natural sequences, we used a fully connected model without any convolutions or recurrent units. The hyperparameters for the *keras* package (version 2.0.8) can be found in its online documentation ([https://keras.rstudio.com](https://keras.rstudio.com/)) [5]. Tensorflow with GPU support was used as the backend. The following hyperparameters were tuned to maximize the average performance on the validation sets:

- network architecture: number of hidden layers, number of nodes per hidden layer, use of dropout layer
- epoch

The following hyperparameters were fixed:

- optimizer = rmsprop
- learning rate = 0.001

The rectified linear ('relu') function was used as the activation function for all layers except the last, which used a sigmoid function. All other hyperparameters were left to their default values. **Logistic regression (LR)** can be represented as a neural network with no hidden layer and was implemented accordingly in *keras.* A fixed optimizer setting incorporating regularization was used to train LR models. The optimized set of hyperparameters for each DNN and LR model is provided in Table B.

**Table B. Hyperparameters for LR and DNN models**

| **Algorithm** | **Dataset** | **Architecture/Hyperparameters** | **Training ACC*** | **Val ACC*^+^** | **Test AUC** | **95% CI Lower** | **95% CI Upper** |
| --- | --- | --- | --- | --- | --- | --- | --- |
| LR | Only Triage | 302 ⇒ 1  optimizer_rmsprop(lr = 0.001), 2 epochs | 0.806 |  | 0.865 | 0.862 | 0.868 |
| LR | Only History | 813 ⇒ 1  optimizer_rmsprop(lr = 0.001), 2 epochs | 0.833 |  | 0.862 | 0.858 | 0.865 |
| LR | Full | 1060 ⇒ 1  optimizer_rmsprop(lr = 0.001), 2 epochs | 0.859 |  | 0.909 | 0.906 | 0.911 |
| DNN | Only Triage | 302 ⇒ 300 ⇒ 100 ⇒ 1  optimizer_rmsprop(lr = 0.001), 3 epochs | 0.814 | 0.812 | 0.873 | 0.87 | 0.876 |
| DNN | Only History | 813 ⇒ 300 ⇒ 30 ⇒ 1  optimizer_rmsprop(lr = 0.001), 4 epochs | 0.845 | 0.841 | 0.872 | 0.869 | 0.876 |
| DNN | Full | 1060 ⇒ 30 ⇒ 30 ⇒ 30 ⇒ 1  optimizer_rmsprop(lr = 0.001), 5 epochs | 0.872 | 0.869 | 0.920 | 0.917 | 0.922 |

* *keras* optimizes accuracy rather than AUC.

^+^ Validation accuracies were not calculated for logistic regression since no hyperparameter tuning was performed.

**Testing the benefit of additional training samples**

Given the large number of independent models needing to be trained on increasing fractions of the training set, two predefined sets of hyperparameters, one set representing a low-complexity model and one representing the previously optimized hyperparameter set of the full model, were used, and the greater of the two AUCs taken as the test AUC value. Given that the hyperparameters were not fully tuned – especially for models built on lower fractions of the training set – the test AUCs are approximate and represent a lower bound.

**Table C. Test AUCs by dataset proportion by algorithm**

| **Algorithm** | **Dataset Proportion** | **Hyperparameters** | **Test AUC** | **95% CI Lower** | **95% CI Upper** |
| --- | --- | --- | --- | --- | --- |
| LR | 0.01 | 1060 ⇒ 1  optimizer_rmsprop(lr = 0.001), 2 epochs | 0.839 | 0.835 | 0.842 |
| LR | 0.1 | 1060 ⇒ 1  optimizer_rmsprop(lr = 0.001), 2 epochs | 0.910 | 0.907 | 0.912 |
| LR | 0.3 | 1060 ⇒ 1  optimizer_rmsprop(lr = 0.001), 2 epochs | 0.910 | 0.908 | 0.913 |
| LR | 0.5 | 1060 ⇒ 1  optimizer_rmsprop(lr = 0.001), 2 epochs | 0.910 | 0.907 | 0.913 |
| LR | 0.8 | 1060 ⇒ 1  optimizer_rmsprop(lr = 0.001), 2 epochs | 0.909 | 0.906 | 0.912 |
| LR | 1 | 1060 ⇒ 1  optimizer_rmsprop(lr = 0.001), 2 epochs | 0.908 | 0.905 | 0.911 |
| XGBoost | 0.01 | max_depth = 10, nrounds = 30,  colsample_by_level = 0.1 | 0.901 | 0.898 | 0.904 |
| XGBoost | 0.1 | max_depth = 20, nrounds = 30,  colsample_by_level = 0.05 | 0.918 | 0.916 | 0.920 |
| XGBoost | 0.3 | max_depth = 20, nrounds = 30,  colsample_by_level = 0.05 | 0.921 | 0.919 | 0.924 |
| XGBoost | 0.5 | max_depth = 20, nrounds = 30,  colsample_by_level = 0.05 | 0.924 | 0.921 | 0.926 |
| XGBoost | 0.8 | max_depth = 20, nrounds = 30,  colsample_by_level = 0.05 | 0.924 | 0.922 | 0.927 |
| XGBoost | 1 | max_depth = 20, nrounds = 30,  colsample_by_level = 0.05 | 0.925 | 0.922 | 0.927 |
| DNN | 0.01 | 1060 ⇒ 30 ⇒ 30 ⇒ 1  optimizer_rmsprop(lr = 0.001), 5 epochs | 0.885 | 0.882 | 0.888 |
| DNN | 0.1 | 1060 ⇒ 30 ⇒ 30 ⇒ 1  optimizer_rmsprop(lr = 0.001), 5 epochs | 0.909 | 0.906 | 0.912 |
| DNN | 0.3 | 1060 ⇒ 30 ⇒ 30 ⇒ 1  optimizer_rmsprop(lr = 0.001), 5 epochs | 0.917 | 0.914 | 0.919 |
| DNN | 0.5 | 1060 ⇒ 30 ⇒ 30 ⇒ 30 ⇒ 1  optimizer_rmsprop(lr = 0.001), 5 epochs | 0.919 | 0.916 | 0.921 |
| DNN | 0.8 | 1060 ⇒ 30 ⇒ 30 ⇒ 30 ⇒ 1  optimizer_rmsprop(lr = 0.001), 5 epochs | 0.919 | 0.917 | 0.922 |
| DNN | 1 | 1060 ⇒ 30 ⇒ 30 ⇒ 30 ⇒ 1  optimizer_rmsprop(lr = 0.001), 5 epochs | 0.921 | 0.919 | 0.923 |

**References**

1. Friedman J. Greedy Function Approximation: A Gradient Boosting Machine. Annals of Statistics. 2000. pp. 1189–1232. Available: http://citeseerx.ist.psu.edu/viewdoc/summary?doi=10.1.1.29.9093

2. Chen T, Guestrin C. XGBoost: A Scalable Tree Boosting System. ArXiv160302754 Cs. 2016; 785–794. doi:10.1145/2939672.2939785

3. Jain AK, Mao J, Mohiuddin KM. Artificial neural networks: a tutorial. Computer. 1996;29: 31–44. doi:10.1109/2.485891

4. Glorot X, Bordes A, Bengio Y. Deep Sparse Rectifier Neural Networks. PMLR. 2011. pp. 315–323. Available: http://proceedings.mlr.press/v15/glorot11a.html

5. Arnold TB. kerasR: R Interface to the Keras Deep Learning Library. In: The Journal of Open Source Software [Internet]. 22 Jun 2017 [cited 2 Feb 2018]. doi:10.21105/joss.00296
